# The Recombinant Human Erythropoietin Administered in Neonatal Rats After Excitotoxic Damage Induces Molecular Changes in the Hippocampus

**DOI:** 10.3389/fnins.2019.00118

**Published:** 2019-02-19

**Authors:** Martha Catalina Rivera-Cervantes, José Jaime Jarero-Basulto, Justo Murguía-Castillo, Alejandra Guadalupe Marín-López, Yadira Gasca-Martínez, Sergio Cornelio-Martínez, Carlos Beas-Zárate

**Affiliations:** ^1^Cellular Neurobiology Laboratory, Department of Cellular and Molecular Biology, CUCBA, University of Guadalajara, Zapopan, Mexico; ^2^Regeneration and Neural Development Laboratory, Department of Cellular and Molecular Biology, CUCBA, University of Guadalajara, Zapopan, Mexico

**Keywords:** monosodium glutamate, erythropoietin, erythropoietin receptor, beta common receptor, recombinant human EPO, excitotoxicity, hippocampus, astrocytes reactive

## Abstract

*In vitro* and *in vivo* experimental evidence has contributed important knowledge regarding the antiapoptotic effect mediated by EPO signaling in the damaged brain, particularly through different models with a hypoxic component. However, little emphasis has been placed on the effectiveness of rhEPO administration against cellular alterations caused by *in vivo* excitotoxicity or on the molecular mechanism that regulates this effect. In this study, we investigated the effects of a single dose of rhEPO on hippocampal damage induced by subcutaneous application of monosodium glutamate (MSG) on postnatal days 1, 3, 5 and 7 in neonatal rats. We found that a dose of 1000 IU/kg of b.w. administered 24 h after MSG had the greatest protective effect. In addition, we analyzed changes in gene expression, particularly in 3 key molecules involved in EPO-mediated signaling (EPO, EPOR and βcR). We observed that the expression of EPO and EPOR was differentially modified at both the mRNA and protein levels under the evaluated conditions, while the expression of the βcR gene was substantially increased. Our data suggest that a low dose of rhEPO is sufficient to induce cellular protection under these experimental conditions and that the molecular changes could be a positive feedback mechanism, mediated by reactive astrocytes in association with *in vivo* neuroprotective mechanisms.

## Introduction

Under physiological conditions, activation of the glutamate receptor (GluR) by its own ligand glutamate (Glu) leads to stimulation of neurons and glial cells; nevertheless, its deregulation is involved in abnormal pathological processes in the brain. Various lines of evidence support the idea that in several neurological diseases, extracellular Glu accumulation overactivates GluR and induces excitotoxic cell death (Meldrum, 2000; Fan and Raymond, 2007; Besancon et al., 2008; Dabrowska et al., 2017). Additionally, the apoptotic cell death that occurs during normal development and/or under pathological conditions shares similar morphological features and a great variety of signaling cascades that induce both of biochemical and molecular events that produce neuronal damage; particularly in susceptible regions such as the hippocampus (Arundine and Tymianski, 2004; Rivera-Cervantes et al., 2004, 2015, 2017; Kwak and Weiss, 2006; Forder and Tymianski, 2009; Zhou et al., 2017). Accordingly, an understanding of the mechanisms that activate these events could allow the design of therapeutic strategies more efficient than the existing ones.

It is recognized that the pathogenesis of many diseases is focal tissue injury, which is triggered by excitotoxicity, oxidative stress and/or inflammatory processes, but effective treatment for this type of injury is lacking (Barker-Haliski and White, 2015; Minhas et al., 2016; Mahaman et al., 2018). To address this issue, we used a neonatal rat model of excitotoxicity model in neonatal rats, a reproducible model whose death rate is only 10% and whose characteristic histopathological and molecular changes have been widely studied. Moreover, this model reproduces the characteristics of a degenerative damage in brain areas associated with the establishment of long-term neurological diseases that present an excitotoxic component. The development of new neuroprotective drugs against Glu excitotoxicity, especially for preventing specific molecular events that trigger cell death, is now an important research goal. Currently, there are few options to approach this problem; however, some molecules, such as erythropoietin (EPO), have great potential as therapeutic agents.

Erythropoietin is a hematopoietic cytokine (glycoprotein), with approximately half its molecular weight being composed of sugar moieties that protect it from degradation. The mature circulating protein contains 165 amino acids and weighs between 30 and 39 kDa (Fisher, 2010). Classically, the action of EPO through binding to its specific membrane receptor (EPOR) regulates red blood cell production by preventing apoptosis of erythroid progenitor cells in response to hypoxia (Rainville et al., 2016; Korzeniewski and Pappas, 2017). For EPO to function in a paracrine manner, tissue must express both EPOR and EPO (Grasso et al., 2004). EPOR and EPO are expressed constitutively and differentially across the embryonic, fetal, and adult stages in the brains of rats, mice, monkeys and humans (Marti et al., 1996; Chin et al., 2000; Dame et al., 2000, 2001). The main neuroprotective function of EPO is believed to be inhibition of apoptosis in cells expressing EPOR (Buemi et al., 2003), which has been detected not only in erythroid cells but also in neurons, astrocytes, microglia and endothelial cells (Nagai et al., 2001; Noguchi et al., 2007; Sargin et al., 2011). In the last few decades, scientific interest in the roles of EPO and EPOR has been focused on the central nervous system (CNS) because it is known that EPOR signaling is required for normal brain development as well as in the restoration of neuronal function after brain damage.

The evidences has shown that not only stressors with a hypoxic component but also other stressors in the brain, such as neurotoxicity and hypoglycemia, are able to induce changes in the expression level of EPO (Morishita et al., 1997; Brines and Cerami, 2008; Kristensen et al., 2010; Yamada et al., 2011; Kastner et al., 2012; Nguyen et al., 2014; Rainville et al., 2016; Nekoui and Blaise, 2017). Although EPO gene expression in the brain is also induced by activation of hypoxia-inducible factors, EPO is not the primary inducer of EPOR (Chin et al., 2000; Grasso et al., 2004); therefore, the regulatory mechanisms of both the production and the processing of EPOR transcripts are uncertain, even under hypoxic conditions.

On the other hand, some studies have shown that the EPO can act through the classical EPOR homodimer (Korzeniewski and Pappas, 2017), but with different affinity: the ligand has a substantially lower affinity for the neuronal-type receptor than for the homodimer, formed by two EPOR subunits (Livnah et al., 1999; Brines and Cerami, 2008). Recent evidence indicates that an EPOR-subunit can physically interact with other cytokine receptors. One candidate is the subunit shared by the granulocyte-macrophage colony stimulating factor, IL-3, and IL-5 receptors, named the β common receptor (βcR), since it is expressed in nonhematopoietic cells and may form a heteromeric receptor with the classic EPOR, playing a specific role in neuroprotection (Brines et al., 2004; Brines and Cerami, 2008).

Additionally, experimental evidence suggests that EPO or even recombinant human erythropoietin (rhEPO) administered exogenously has a neuroprotective effect against CNS injuries. This molecule can act in a coordinated fashion in multiple protective pathways, including inhibition of apoptosis, restoration of vascular autoregulation, attenuation of inflammatory responses, and restorative functions, through stem-cell recruitment (Eid and Brines, 2002; Grasso et al., 2004; Gyetvai et al., 2017; Li et al., 2018).

Although there is a substantial amount of evidence regarding the multiple effects associated with EPO signaling in the brain, relatively little is known about its potential role in excitotoxicity in the brains of neonatal rats or the mechanism of production and regulation of molecular markers of EPO signaling, and even less is known about its effects *in vivo* during postnatal development of the brain.

For the above reasons, the aim of this study was to investigate the expressional changes of signaling molecules through which rhEPO could achieve its neuroprotective effect in the hippocampus of neonatal rats with excitotoxic brain injury induced by MSG administration (Rivera-Cervantes et al., 2009; Rivera-Carvantes et al., 2017). With this finality, we first established the optimally efficient dose of rhEPO with the greatest neuroprotective effect and then evaluated the changes in gene expression levels induced by this molecule, particularly in key molecules at the onset of EPO-mediated signaling, such as EPO, EPOR and the postulated βcR. To harness the neuroprotective power of EPO, we must first understand the EPO/EPOR system and its regulation; however, the signaling pathways triggered by this stimulus are not completely understood yet.

## Materials and Methods

The following reagents were used, monosodium L-glutamate (MSG-G1626 from Sigma Chemical Co., St. Louis, MO, United States); RNeasy^®^ Plus Mini Kit (Catalog number 74134, QIAGEN, Hilden, Germany), Gotaq probe qPCR Master Mix 2X (A610A, Promega, Madison, WI, United States), M-MLV Reverse Transcriptase (M-MLV28025-021, Promega), and random primer (1575927 Invitrogen, Waltham, MA, United States), goat polyclonal antibody to EPOR (1:1000, Sc-82590 Santa Cruz, Biotechnology, INC., Dallas, TX, United States) or rabbit polyclonal antibody to β-actin (1:1000, 8457, Cell Signaling Technology, Danvers, MA, United States) and for immunofluorescence was utilized rabbit polyclonal antibody anti-GFAP (1:1000; ab7260, Abcam, Cambridge, United Kingdom). While that the recombinant human erythropoietin rhEPO (Exetin-A) was purchased of Pisa Pharmaceutical, México. All other chemicals used were of the highest purity that was locally available.

### Animals and Treatments (MSG and rhEPO)

Newborn male Wistar rats were housed under temperature-controlled conditions (22 ± 2°C) and a relative humidity (50 ± 4%) with 12 h light-dark cycle. Water and food were freely available. All litters were reduced to 7–8 pups per female at birth to standardize conditions. All experimental procedures were performed in accordance with the ethical rules and were approved by the Institutional Ethical Committee according to the Mexican General Health Laws and their corresponding chapters in 1987 and the National Institutes of Health Guidelines for the Care and Use of Laboratory Animals (NIH Publication No. 80.23, revised 1996 and were in accordance with the Mexican Official Norms (NOM-062-ZOO-1999 and NOM-033-ZOO-1995) and with the Directive 2010/63/EU referenced rules. Furthermore, the Guadalajara University’s Bioethics Committee (México) approved the experimental protocols (CC/NN 11-12/00/2012). All efforts were made to minimize the number of animals used and their suffering.

We used the MSG model previously described in other works (Rivera-Cervantes et al., 2009). Briefly, excitotoxicity was induced via subcutaneous (s.c.) administration of four doses of MSG [4 mg/g of body weight (b.w.)] at PD 1, 3, 5, and 7. To determine the effective dose of neuroprotection, in the corresponding experimental groups were administered one of the different doses of rhEPO (250, 500 or 1000 IU/kg of b.w., via s.c.), and then in the PD 8 were analyzed through to histological studies. Once established the dose of neuroprotection, only was used a doses of EPO 1000 IU/kg of b.w for molecular analysis. Two additional groups were used as controls the first group of untreated animals was used as *Intact Group* for all studies addressed, while that a second group was treated only with rhEPO (without MSG). We used an n = 4 per each study.

### Histological and Immunofluorescence

#### Histological Studies

The brain tissues were isolated from all study groups (control and experimental). The rats were anesthetized by intraperitoneal injection of sodium pentobarbital (50 mg/kg of b.w.) and perfused transcardially with fixative solution (4% paraformaldehyde, in 0.1 M PBS, pH 7.2) at PD 14. All fixed brains were coronally sectioned into 2.5 cm slice blocks and embedded in paraffin; 8 μm slices were obtained to each brain and then stained with cresyl violet. Stained samples were visualized with a 40X (NA 0.75) dry Plan Fluor objective on a Nikon ECLIPSE 55i light microscope (Nikon Corp.; Tokyo, Japan). Images of the CA1 hippocampal area were captured and analyzed with the Nikon NIS-Elements software by an experimenter blind to the treatments that the animals had received. For quantification, six sections of each brain in each group of animals were studied (n = 4) in six fields per section (bilateral). The results were expressed as the average over the experimental “n”, which was the number of normal cells counted and averaged between the cell counts in the hippocampus bilaterally.

#### Immunofluorescence Staining and Digital Image Analysis

Sections from the same experimental groups were processed for double immunolabeling using protocols described in previous works (Jarero-Basulto et al., 2013) to determine the degree of glial activation by the excitotoxic effect of MSG. Briefly, the perfused brain was excised and postfixed in 10% sucrose for 72 h at 4°C. Subsequently, the tissue was frozen and cut into serial coronal sections (20 μm) using a cryostat (CM1860 Leica, Wetzlar, Germany). Sections were washed with phosphate-buffered saline (1X PBS) and then incubated in 1% nonfat dry milk in PBS with 0.2% Triton X-100 (PBS-T) at room temperature (RT) for 20 min. For GFAP staining, the sections were incubated with rabbit polyclonal antibody anti-GFAP (1:1000; Abcam) overnight at 4°C. Then, the sections were extensively washed in PBS-T and incubated for 2 h at RT with fluorescein isothiocyanate-tagged anti-rabbit secondary antibody (1:200; 115-095-008, Jackson ImmunoResearch Laboratories, Inc., West Grove, PA, United States). In the case of double labeling, at the end of the procedure, the sections were mounted using anti-quenching Vecta-shield medium with DAPI (H-1200, Vector Laboratories, Inc., Burlingame CA, United States) to visualize the nucleus.

The immunolabeled sections from the hippocampus were viewed and analyzed using a DMI8 fluorescence microscope (Leica, Wetzlar, Germany). Samples were observed using a 40X (NA.1.25-0.75) objective. The GFAP-positive cells in all study groups were quantified from the resulting images using Leica DMI8 software (LAS V4.12.InK). These data were plotted as the number of cells positive for GFAP with respect to the study group for a count of three fields per section bilaterally, and the number of total cells was averaged for *n* = 4 experiments.

### Molecular Studies

According to the histological data obtained, we determined that at least in these experimental conditions, the effective protective dose was 1000 IU/kg of b.w.; for that reason, this dose was used for the following experimental procedures (both mRNA- and protein-level gene expression). The animals in this study were sacrificed at 6 and 24 h after rhEPO (for the intact and MSG groups, the animals were sacrificed at PD 8 and 9) for RT-PCR, while for immunoblot study the animals were sacrificed 24 h after rhEPO administration. Unfixed samples of hippocampi were collected from each study group and dissected out at 4°C for molecular studies.

#### Real-Time RT-PCR

Total RNA was extracted from the rat hippocampal tissues using the RNeasy Plus Mini Kit (74134, QIAGEN). RNA quality and quantity were assessed in a microplate spectrophotometer (Thermo Fisher Scientific, Waltham, MA, United States), followed by first-strand cDNA synthesis using M-MLV Reverse Transcriptase (M-MLV28025-021, Promega) enzyme and random primers (1575927, Invitrogen) at 70°C for 5 min, 37°C for 60 min. RT-PCR analyses were performed in 96-well PCR plates using TaqMan probes for genes Rplp1 (179767531), Gapdh (179767533), β-Actin (179767525), EPO (179767532), EPOR (179918870) and βcR (206427008) [all from Integrated DNA Technologies (IDT), Coralville, IA, United States] with 2 μL of cDNA, 10 μL of Go-Taq probe PCR Master Mix 2X (A610A, Promega), 1 μL of TaqMan probe 20X and 2 μL of nuclease-free water. Reactions were run on an RT-PCR StepOne Plus system 4376592 (Applied Biosystems, Foster City, CA, United States) under the following conditions: the amplification started at 95°C for 2 min, followed by 40 cycles of 95°C for 15 s and 60°C for 1 min. Each biological replicate was analyzed in technical triplicate. Normalization was performed using the rat Rplp1 gene, which was the housekeeping gene with the least variation between groups. Amplification results from average cycle threshold (Ct) data were processed using the 2^−ΔΔCt^ formula and expressed as a fold up- or downregulation difference/change between experimental conditions. All data below 1 were transformed to negative by [(−1)/value below] formula.

#### Immunoblots

##### Obtained protein extract

Frozen samples (−80°C) of the hippocampus from the different study groups were processed for the isolation of proteins. Proteins from brain tissue were obtained by extraction with a Mem-PER^TM^ Plus kit (89842, Thermo Scientific). The hippocampus samples, once defrosted, were washed in buffer solution for 15 s; then, each sample of tissue was homogenized at 4°C in 500 μL of permeabilization buffer, after which 250 μL of buffer was added, and the mix was incubated for 10 min at 4°C in gentle shaking. The homogenates were centrifuged at 16,000 X g for 15 min at 4°C, and the supernatant (containing cytosolic proteins) was recovered carefully and transferred to a new tube. For the other part, the pellet was resuspended in 300 μL of solubilization buffer and incubated for 30 min at 4°C in shaking. After centrifugation at 16,000 X g for 15 min, the supernatant was recovered and transferred to a new tube containing membrane-associated proteins. Homogenates from both fractions were quantified on a microplate spectrophotometer at 595 nm (Thermo Scientific) using the Bradford protein assay reagent (Sigma-Aldrich) and a bovine serum albumin (BSA) standard. Homogenates were aliquoted and stored at −20°C until the day of the experiment.

##### Dot blot

The membrane and cytosolic protein fractions were analyzed by dot blot under nondenaturing conditions as previously described (Jarero-Basulto et al., 2013). Using a Bio-Dot microfiltration apparatus (Bio-Rad), 40 μL of each fraction of proteins was spotted onto nitrocellulose membranes and then blocked with 10% nonfat dry milk in PBS 1% Triton (PBS-T) for 1 h at RT with gentle shaking. After being washed in PBS-T, spotted membranes were independently incubated with either goat polyclonal antibody against EPO (1:1000, BIOSS), goat polyclonal antibody against EPOR (1:1000, Santa Cruz Biotechnology) or rabbit polyclonal antibody against β-actin (1:1000, Cell Signaling) for 40 min at RT with gentle shaking. After being washed in PBS-T, the membranes were incubated with the corresponding HRP-tagged anti-goat secondary antibodies (EPOR) and rabbit IgG (EPO and β-actin) (1:20000) in PBS-T for 40 min at RT. Reactivity was visualized using the WesternSure Premium substrate (LI-COR) and imaged for 1 min on Carestream autoradiography plaques. The dot blots were quantified using the ImageJ program.

### Statistics

Statistical comparisons were performed using a one-way analysis of variance (ANOVA), and Tukey’s HSD test was used for the post hoc comparisons between groups. *P* < 0.05 was considered significant. Data are shown as the means ± standard errors of the means (SEM), for both protein-expression and histological study.

## Results

### rhEPO Administered in a Single Dose Has an Efficient Neuroprotective Effect Against the Excitotoxic Damage Induced by MSG in the Hippocampus of Neonatal Rats

To determine the single dose of rhEPO that induced the greatest cellular protection against damage induced by MSG, we carried out a quantitative analysis of the number of cells with normal morphology evaluated by Nissl staining in the CA1 hippocampal area of the brains of the rats from different experimental groups.

**FIGURE 1:**
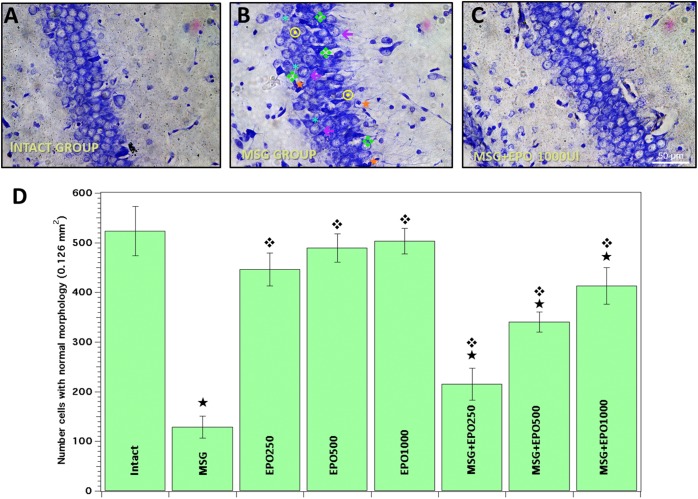
rhEPO treatment diminishes the cell damage in rat CA1 hippocampal region, caused by MSG. Evaluation by Nissl staining was performed to all study groups. **(A)** Normal cells with intact morphology were commonly observed in intact group slices. **(B)** Significant morphological alterations were evident in the same region in MSG group [perikarion atrophy 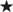, pyknotic nuclei 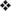, basophilia ddd118, cytoplasmic retraction ^∗^, increase cellular bulk ←]. **(C)** More density of cell with normal morphology was observed in MSG + EPO (1000 IU/kg b.w.) group (representative photomicrograph). All the images in panels **(A–C)** were obtained from a CA1 area of hippocampal region. Scale bars = 50 μm. **(D)** Total number of cells with normal morphology in all experimental groups, ^
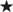
^significant value vs. intact group, significant value vs. MSG group; all bars represent the average ± standard deviation.

In the light-microscopy analysis of sections (particularly of the CA1 area) from the brains of MSG-treated rats (MSG group), neurons showed characteristic damage, such as cytoplasmic retraction, basophilia, perikaryon atrophy, pyknotic nuclei, partial or total loss of dye affinity or increase in cellular bulk (Figure 1B), and only a few isolated normal cells were evidenced at PD 14. In contrast, when we evaluated the hippocampi of both the control untreated group (intact group) and the groups treated with rhEPO only at different doses (250, 500 or 1000 IU/kg of b.w.) (EPO groups), we never observed cellular abnormalities in this region (Figure 1A, representative photomicrography of the intact group).

We also compared the MSG group to each experimental group treated with a single dose of rhEPO (250, 500 or 1000 IU/kg of b.w.) Twenty-four hours after the MSG treatment (MSG + EPO groups), we observed that the evidence of morphological alterations decreased in a dose-dependent manner (Figure 1C, representative image of 1000 IU dose), and the number of normal cells was elevated with respect to that in the MSG group (Figure 1B).

To validate the contribution to each single dose of rhEPO in the neuroprotective effect against MSG treatment, we quantified and compared the numbers of normal cells among the group studied. In Figure 1D, quantitative analysis was plotted as the numbers of normal cells in the control groups (intact and EPO 250, 500 and 1000), between which no statistically significant differences were registered. However, in the CA1 hippocampal region of neonatally MSG-treated animals, 75% cellular loss was registered with respect to the quantity in the same region of the intact group. When rhEPO was administered 24 h after the final MSG treatment, we observed a dose-dependent effect on cell damage restoration upon quantitative analysis of the cells with characteristic normal morphologic morphology when compared with the CA1 region of the brain in the MSG group. As shown in Figure 1D, increased numbers of cells (66, 163 and 218%) were found when rhEPO was administered at doses of 250, 500 and 1000 IU/kg of b.w., respectively, compared to the same brain region in the MSG group.

The principal differences were obtained when rhEPO (1000 IU/kg of b.w.) was administered after MSG. This dose was utilized for the evaluation of glial reactivity (astrocytic response) by immunofluorescence and for the evaluation of changes in both protein and gene expression of EPO, EPOR and βcR using real-time PCR (RT-PCR) in subsequent experiments.

### rhEPO Treatment Reduces the GFAP Immunoreactivity Induced by MSG

Because glial activation is a normal response to neuronal damage, we decided to investigate glial activation in the CA1 hippocampal area after MSG application and whether it was affected by the administration of a single dose of rhEPO (1000 IU/kg of b.w.) using double-label immunostaining with an antibody against glial fibrillary acidic protein (GFAP) in combination with the nucleus-specific DAPI dye (green and blue markers, respectively).

**FIGURE 2:**
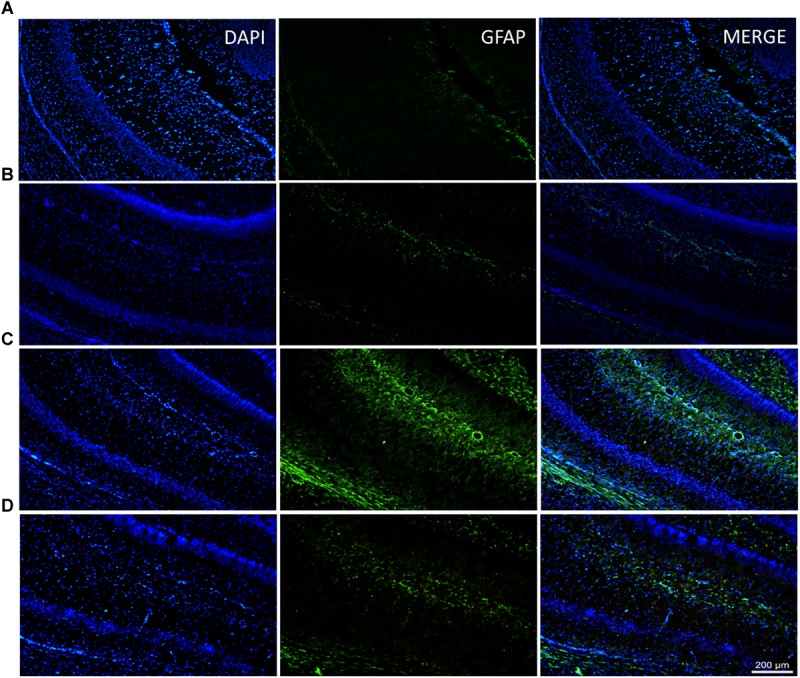
Diminish of the glial response in the CA1 hippocampal area after rhEPO administration. Brain slices of all study groups were processed for double labeling epifluorescence by combining anti-GFAP (to astrocytes) and DAPI (to nuclear stain) and analyzed by epifluorescence microscopy. **(A–D)** Examples of labeled astrocytes are indicated in the medium panel (green channel), the left panel represents nuclei stain (blue channel) and right only display the merge channel from their corresponding CA1 fields. **(D)** In the MSG + EPO group, GFAP show a less reactivity compared with MSG group **(C)**. Scale bars = 200 μm.

**FIGURE 3:**
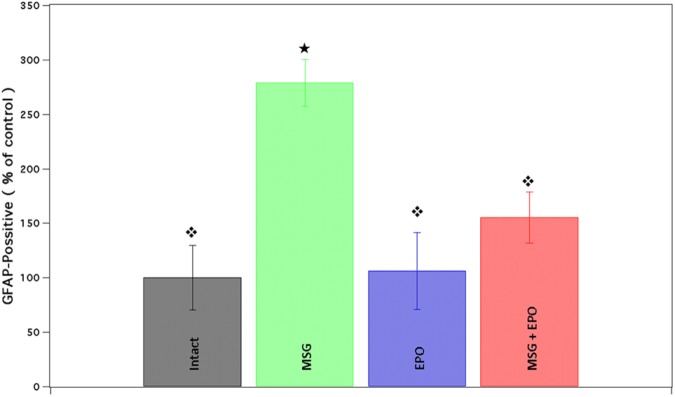
The percentage from astrocytic cells in the CA1 brain area is dramatically diminished after rhEPO administration. The percent of GFAP positive cells (per three fields) was obtained and compared with all study groups (Intact, EPO, MSG and MSG + EPO). In the MSG + EPO group, significantly less number of astrocytes was found vs. those of MSG group (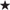), but similar at control groups.

The hippocampi of MSG group samples showed elevated glial activation with an increased number of processes (Figure 2C) compared with the control groups (intact or EPO), which presented only basal immunoreactivity (Figure 2A,B, respectively). Comparatively, in the MSG + EPO 1000 group (Figure 2D), GFAP-positive immunoreactivity was observed, although this was decreased considerably with reference to the hippocampi of the MSG group, it was slightly elevated with respect to the hippocampi of the control groups. These data were further confirmed by counting the number of GFAP-positive cells in tissue sections of the hippocampus (CA1 area) in each experimental group (Figure 3). The graph shows that no significant differences were registered between the numbers of GFAP-positive cells in the intact and EPO 1000 groups, in contrast to the MSG-treated rats, in which a substantial increase in GFAP-positive cells was observed, while a dose of rhEPO (1000 IU/kg, b.w.), after MSG treatment, induced a decline in immunoreactivity to GFAP, although it did not reach the control (intact group) levels observed (Figure 3).

### Gene Expression Levels of EPO, EPOR and βcR Are Modified by rhEPO Treatment Under Excitotoxic Conditions

**FIGURE 4:**
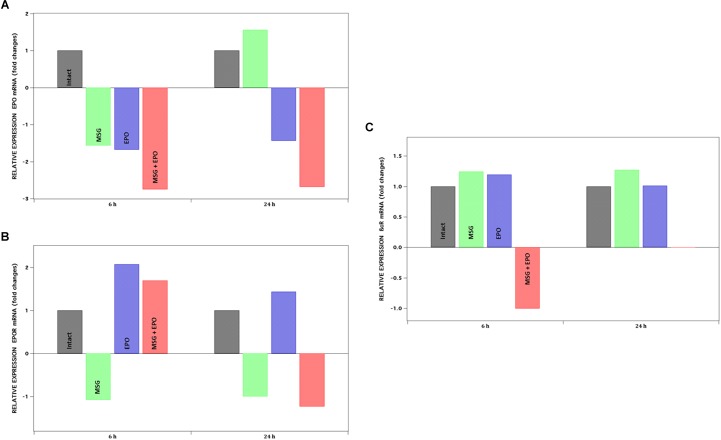
mRNA expression levels of EPO, EPOR, ßcR. Genes were analyzed by qPCR at 6 and 24 h after rhEPO administration, represented at left and right panel of each graph respectively. Y-axis shows the positive or negative fold change for each gene: **(A)** EPO, **(B)** EPOR and **(C)** ßcR. Fold change ± 1.5 is considered as significant value.

Quantitative RT-PCR was used to evaluate a subset of rhEPO-regulated genes. The rhEPO was used at a dose of 1000 IU/kg of b.w. to evaluate the molecular parameters in MSG-treated rats at 6 and 24 h after administration. The data were expressed for all genes as fold changes with respect to the level expression of the control group, normalized to one (Figure 4). Analyzing the changes in the expression levels of all genes in the pharmacological control group (EPO group) at 6 and 24 h, a tendency to increase was registered for both the EPOR and βcR genes, although only significant for the EPOR gene at 6 and 24 h; for the other part, the EPO expression was diminished at both times, as shown in Figure 4.

#### The EPOR Gene Was Induced by rhEPO Treatment Under Excitotoxic Conditions, but no EPO Expression Was Observed

The administration of four doses of MSG to rats at neonatal ages did not induce significant changes in the level of expression in the evaluated gene, except for EPO gen at 24 h (increased in 0.5 fold with respect to intact expression). Moreover, the level of expression of the EPO gene in MSG + EPO-treated rats was lower than that in the intact group at 6 and 24 h (−2.75- and −2.68-fold, respectively) (Figure 4A).

With respect to the changes in the level expression of EPOR mRNA, we registered that treatment with four doses of MSG did not induce significant changes with respect to the expression level in the intact group at both time points evaluated. However, both treatments (MSG + EPO group) induced a significant increase in the level of EPOR mRNA at 6 h post-rhEPO, 2-fold higher than the level of expression registered for the intact group (Figure 4B).

#### Significant Changes in the Expression Level of βcR Gene Was Observed

As seen in the data plotted in Figure 4, the MSG treatment did induce a slight increase (although not significant) in the βcR-gene expression levels in both evaluated times; however, the treatment with MSG and rhEPO induced differentially opposite changes in both evaluated times (6 and 24 h), given that reduced expression (one-fold) of this gene at 6 h was observed, and at 24 h, a substantially significant increase was registered, measuring more than 5000-fold; these data were not plotted because they are out of range of the other values on the graph, although they were reproducible in all the experiments carried out.

### The EPO and EPOR Protein Levels Are Modified Differentially by Both MSG and rhEPO Treatments

To continue with the assessment of the molecular response to excitotoxicity generated by MSG treatment and the cellular protection with a single dose of rhEPO in the hippocampal cells from rats, we decided to investigate whether the expression levels of EPO and EPOR proteins in two cellular fractions (cytoplasmic and membrane) 24 h after rhEPO administration.

**FIGURE 5:**
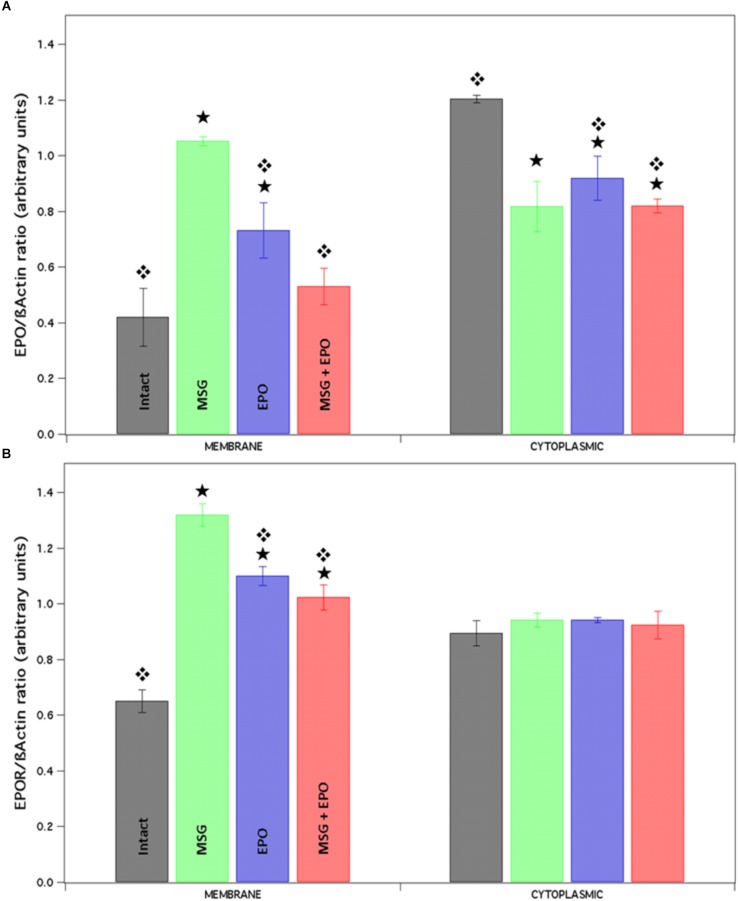
The expression level of proteins EPO and EPOR is differentially modified after rhEPO administration. Native proteins were isolated from the brains of each study group and by each one of them we obtained two different fractions (membrane and cytosolic). Both fractions were spotted and analyzed for Dot blot with EPO and EPOR antibodies. Finally, the results were analyzed using the Image J program and plotted in the graphic. **(A)** The expression of EPO was differently modified in both fractions, lightly high and lightly down respect to control (membrane and cytoplasmic fraction respectively). While, EPOR expression presented a significantly increment in the membrane fraction only respect to the control, none change was observed in a cytoplasmic fraction **(B)**. ^
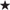
^Statistical differences between intact vs. experimental. ^
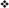
^Statistical differences between MSG vs. experimental groups.

We verified the protein expression by dot blot using whole hippocampal protein extracts (to isolate two fractions), and the immunoreactivity to both EPO and EPOR proteins was analyzed using anti-EPO and anti-EPOR antibodies (see section “Materials and Methods”). In both cases, the plotted data were normalized to the level of a constitutively expressed protein (β-actin, Figure 5). In the case of the EPO protein expression level, we observed a significant increase in the membrane fraction in the MSG and EPO groups and a slight increase in the MSG + EPO group. All of the groups were compared with the control group. Concomitantly, in the cytoplasmic fraction, we detected a reduction in the protein expression levels in all groups with respect to the control but no statistically significant changes between them, were observed (Figure 5A, left and right sides, respectively).

When we analyzed the expression of EPOR in the same samples of proteins into the membrane fraction, we found significant increases in all samples with respect to control expression, but with differences between them, especially for MSG samples; in contrast, the cytoplasmic fraction never showed differences in expression between the study groups (Figure 5B, left and right sides, respectively).

## Discussion

L-Glu is considered the principal excitatory neurotransmitter in the mammalian CNS, and normally, its concentration increase in the extracellular space is limited to a very short period and in a localized region solely for synaptic transmission processes (Watkins and Jane, 2006; Zhou and Danbolt, 2014). Nevertheless, it has been suggested that massive, sustained, nonlocalized changes in L-Glu levels could be responsible for generating abnormal cellular death, events that play a pivotal role in neurodegeneration in the CNS.

Both *in vitro* and *in vivo* evidence have demonstrated that cells exposed to Glu show different processes of death (e.g., apoptosis, autophagy, necroptosis, etc.), and those results seem to depend on the dosage and duration of Glu stimulation, as well as the metabolic state of the cell (Fujikawa, 2015; Jia et al., 2015; Fricker et al., 2018), making its comprehension challenging.

In previous works, our team reported that the administration of four doses of MSG (4 mg/g of b.w., s.c.) in neonatal rats at PD 1, 3, 5 and 7 induces an increase in the level of Glu (Lopez-Perez et al., 2010). The results suggest that this increase is responsible for promoting both molecular and morphological abnormal changes in the cells; some of them have been identified in the short and long term and are associated with injury events, as well as increased susceptibility to seizures in older animals, although conclusive studies are required in order us to confirm whether the model used would be a suitable model of epileptogenesis; for this reason this excitotoxicity model was utilized in the present study. These changes have been widely studied in the hippocampus, a brain region considered to be highly susceptible to damage (Rami et al., 1997).

On the other hand, multiple evidences have confirmed that a dose (500-5000 IU/kg b.w.) of rhEPO administered systemically in rodents, sheep and primates, appears within the cerebrospinal fluid with a mean delay of approximately 1 h, reaching neuroprotective concentrations (Brines et al., 2000; Ehrenreich et al., 2004; Juul et al., 2004; Statler et al., 2007). Based on the above findings, and because rhEPO dose concentration may represent differences in cellular protection, no data about its administration have been reported in this MSG excitotoxic model. Therefore, we decided to establish a single dose of rhEPO with an optimal cell-protective effect against damage in the hippocampus from MSG-treated rats. We administered a subcutaneous dose of rhEPO (250, 500 and 1000 IU/kg of b.w.) 24 h after the last application of MSG. Although the animals were sacrificed at PD 14 for the first objective of the present study, we previously reported massive cellular damage in the evaluated CA1 region by different methods (viability assay and TUNEL and Nissl stain) and in different postnatal ages (Rivera-Cervantes et al., 2009; Rivera-Cervantes et al., 2015; Rivera-Carvantes et al., 2017). Particularly, it is important to mention that in other work, the temporal course of the excitotoxic damage has been reported from day 2 to 14 PD in the same model (Rivera-Cervantes et al., 2015; Rivera-Carvantes et al., 2017). These reports showed that the triggering of cellular damage in the hippocampus by four doses of MSG (s.c.) is slow and progressive. Accord to this, it is evident that a delayed process in excitotoxic cell death could be an option to find a way to prevent or stop cell death and thus diminishes the clinical consequences of damage. For this reason the mechanism of neuroprotection associated with endogenous molecules are of current research interest.

Based on the above findings in the present work, we observed a protective effect of rhEPO administration via s.c. in neonatal rats. This is the first report using a model of excitotoxic damage *in vivo* with MSG. The effect of the administration of rhEPO registered for quantitative analysis of number cells with normal morphology (Nissl stain) was dose-dependent, after to suffer excitotoxic damage with MSG. Similar data have been reported in other studies using kainic acid in an excitotoxic model *in vitro* (Morishita et al., 1997), and other through of modulation of NMDAR activity (Dzietko et al., 2004; Keller et al., 2006). In contrast to other reports in the literature, the present study evaluated relatively low doses of rhEPO, in the range of 250 to 1000 IU/kg of b.w. We found that with a dose of 1000 IU/kg of b.w. has an significant protective effect similar to that observed in other studies in which this effect was reported in the hippocampus of rats treated with MK801, in those studies, however, a dose of 20,000 IU/kg of b.w., of rhEPO was required to protect 50% of the pyramidal neurons in CA1 (Dzietko et al., 2004). These data suggest that the broad efficacy of rhEPO observed in different models depends on its key roles in multiple protective pathways, that induce molecular changes, as well as the age of development and the post-damage time to which the EPO is administered; which determine the tissue response (Marti et al., 2000; Kawakami et al., 2001; Weber et al., 2002; Genc et al., 2004; Spandou et al., 2005).

Once the cytoprotective dose of rhEPO (1000 IU/kg of b.w.) was established, the activation states of astrocytes were evaluated by an analysis of GFAP immunoreactivity in response to excitotoxic damage at PD 14. Astrocytes are activated by inflammatory mediators and produce regulatory factors such as cytokines. Propagation of cytokines and secondary signals leads to physiological and pathological components of the sickness response. The GFAP immunoreactivity increased significantly in the CA1 hippocampal region of rats treated with MSG, as previously reported by Ortuno-Sahagun et al. (2012), although in that study, other hippocampal regions were evaluated at PD 8. The quantitative evaluation showed a significant increase in the number of GFAP-positive cells when MSG was administered, which could be indirectly correlated with the upregulation of both the gene and protein expression levels of inflammatory regulatory such as TNF-α, IL-1β and IL-6 cytokines, as reported by Chaparro-Huerta et al. (2005) in this same excitotoxic condition. These results provide positive feedback that promotes a further increase in the number of GFAP-positive cells, as observed in the CA1 hippocampal region at PD 14; based on the previously mentioned facts, the application of rhEPO in rats with excitotoxic damage could be preventing the production of proinflammatory cytokines, which, in turn, would reduce glial activation.

Moreover, the paracrine role of the EPO/EPOR system in neuroprotection after damaging events, particularly those with a hypoxic component that increases the production of diverse endogenous gene and therefore protein levels of the signaling mediated by EPO through of mechanism previously described in the literature, suggests that EPO may act as an endogenous mediator of neuroprotection and tissue restoration in response to this type of insult (Bernaudin et al., 1999; Chin et al., 2000; Spandou et al., 2004; Tsai et al., 2006).

However, other stress stimuli, such as those that induce the production of proinflammatory cytokines such as TNF, greatly increase EPOR levels (Nagai et al., 2001). To clarify the expression pattern in the rat brain after induced excitotoxic damage, we studied whether rhEPO administration induced the short-term modulation of EPO and EPOR at both the mRNA and protein levels. Previously been no reports of excitotoxicity-induced changes in the signaling mediated by EPO, in the present work, the neonatal administration of MSG increased only when evaluated at 24 h after excitotoxicity in the mRNA level of EPO, but the protein level was increased in both EPO and EPOR protein at 6 h. These notable differences found between mRNA and proteins, as well as between the expression levels found at the two times evaluated, likely do not reflect coordination in the expression of these molecules. As has been probed in the ischemic case, EPOR expression is prior to the EPO gene. However, the observed changes in the present work allow us to suggest that this modification could also be related to endogenous mechanisms of cellular protection, possibly related to the production of TNF (Nagai et al., 2001; Chaparro-Huerta et al., 2005). We found that the administration of rhEPO alone induced a significant increase in EPOR mRNA in the evaluated time period, while an increase in both EPO and EPOR proteins was observed. Similarly, in the hippocampi treated with MSG and rhEPO (MSG + EPO group), the expression level of EPOR increased, but, as observed in the experimental conditions evaluated, only some of the changes presented similar behavior between mRNA and protein expression levels. These differences could be explained by the fact that mRNA transcripts of both EPO and EPOR have limited intracellular stability; EPOR mRNA has a half-life of only 90 min in human erythroid progenitor cells (Wickrema et al., 1992), in contrast to other studies where the expression changes, principally in EPOR, in response to both excitotoxic (Morishita et al., 1997) and ischemic damage (Bernaudin et al., 1999) were not reported until 4 h after injury. Since the increased levels of EPO and EPOR were recorded at 6 h after rhEPO administration in the present study, it is possible that the production and maturation mechanisms of the proteins are different and depends on the trigger stimulus.

Moreover, these results are in agreement with other studies in which an increase in the expression level of these markers was reported as a result of the induction-damage (Wickrema et al., 1992; Bernaudin et al., 1999). Additionally, the expression changes of EPO and EPOR under the conditions mentioned above are in agreement with the autoregulation mechanism mediated by endogenous EPO or rhEPO application (Komatsu et al., 1997; Wallach et al., 2009). Although such changes are not completely understood, especially in the CNS, it has been postulated that rhEPO induces changes in the mRNA expression of both EPO and EPOR. Nonetheless, it may be difficult to discern what proportion of the observed effect is a result of differential susceptibility to excitotoxic damage in neonatal life and whether these changes are mediated by the administered rhEPO.

Finally, although no evidence exists in the literature that rhEPO treatment promotes a change in the expression of βcR under the present conditions, we observed a significant increase in the level of its gene transcript in the hippocampus, both at 12 h and at 24 h, with very high levels at the last time point evaluated in the MSG + EPO group (see “Significant changes in the expression level of βcR gene was observed” in Results section). These data could support the hypothesis of Brines and Cerami, 2008, related to the participation of this subunit of βcR in the mechanism of protection mediated by rhEPO, particularly in the CNS, although the molecular pathway for which this occurs should be evaluated in future studies.

A possible explanation of the mechanism involved in the molecular changes observed in the work for the effect of application of four doses of MSG in neonatal rats is centered on the fact that this damaging event does not include a hypoxic component. As has been reported under these conditions, increases in the expression levels of cytokines and inflammatory mediators, as well as in astrocytic reactivity, could be determinants of the biological response of the CNS. A significant increase in GFAP immunoreactivity induced by excitotoxicity is maintained from PD 8 (Ortuno-Sahagun et al., 2012) to PD 14, as is shown in the present work. The above findings suggest that molecular changes could be a positive feedback mechanism mediated by reactive astrocytes; however, it will be necessary to conduct future experiments using other methods that examine the colocalization of these proteins, since different studies demonstrate that different inflammatory regulators are induced by glial reactivity (Carpentier et al., 2005).

## Conclusion

In conclusion, our data suggest that a single dose of rhEPO is sufficient to induce cellular protection in the hippocampus of neonatal rats treated with four doses of MSG. We also provide evidence that regulation of EPOR, EPO, and even βcR expression occurs under excitotoxic conditions, although the effect is different for each gene and protein and at each evaluated time, being most prominent (particularly at the protein level) when rhEPO is administered 24 h after MSG. In summary, we suggest that this could be a positive feedback mechanism mediated by reactive astrocytes; however, it will be necessary to conduct future experiments using other methods that examine the colocalization of these proteins, allowing us to confirm the relevance of these findings in association with *in vivo* neuroprotective mechanisms.

## Author Contributions

MR-C and JJ-B conceived and designed the study, acquired the data, or analyzed and interpreted the data, the contribution of two first authors, is the same level of importance. JM-C and AM-L critically reviewed all intellectual content and data processing. SC-M provided the technical support. CB-Z reviewed and approved the final version to be submitted.

## Conflict of Interest Statement

The authors declare that the research was conducted in the absence of any commercial or financial relationships that could be construed as a potential conflict of interest.
